# Recent Advances in Chitin and Chitosan/Graphene-Based Bio-Nanocomposites for Energetic Applications

**DOI:** 10.3390/polym13193266

**Published:** 2021-09-25

**Authors:** Rabia Ikram, Badrul Mohamed Jan, Muhammad Abdul Qadir, Akhmal Sidek, Minas M. Stylianakis, George Kenanakis

**Affiliations:** 1 Department of Chemical Engineering, University of Malaya, Kuala Lumpur 50603, Malaysia; 2 Institute of Chemistry, University of the Punjab, Lahore 54590, Pakistan; mabdulqadir@gmail.com; 3 Petroleum Engineering Department, School of Chemical and Energy Engineering, Faculty of Engineering, Universiti Teknologi Malaysia, Johor Bahru 81310, Malaysia; akhmalsidek@utm.my; 4 Institute of Electronic Structure and Laser, Foundation for Research and Technology-Hellas, N. Plastira 100, Vasilika Vouton, GR-700 13 Heraklion, Greece; stylianakis@iesl.forth.gr

**Keywords:** polymers, graphene oxide, bio-nanocomposites, chitosan, energy

## Abstract

Herein, we report recent developments in order to explore chitin and chitosan derivatives for energy-related applications. This review summarizes an introduction to common polysaccharides such as cellulose, chitin or chitosan, and their connection with carbon nanomaterials (CNMs), such as bio-nanocomposites. Furthermore, we present their structural analysis followed by the fabrication of graphene-based nanocomposites. In addition, we demonstrate the role of these chitin- and chitosan-derived nanocomposites for energetic applications, including biosensors, batteries, fuel cells, supercapacitors and solar cell systems. Finally, current limitations and future application perspectives are entailed as well. This study establishes the impact of chitin- and chitosan-generated nanomaterials for potential, unexplored industrial applications.

## 1. Introduction

In recent decades, nanotechnology advancements have led towards the progressive recycling of natural polymers into a variety of structurally enhanced nanomaterials [1,2]. This has been proven by the synthesis of polymer-based carbon nanomaterials (CNMs) [3]. The implication of CNMs has been established through various fields, such as biological activities [4], drug delivery [5], tissue engineering [6], environmental [7] or energetic applications [8]. Their unique properties have allowed them to be used in several electronic devices [9]. It has been observed that dipole–dipole interactions between molecules and powerful van der Waals forces have produced aggregation among CNMs [10]. Fortunately, the consequences of this limitation, such as the modification of electrical, chemical and mechanical properties, have been modified through functionalization [11]. Graphene is an excellent example of a CNM due to its remarkable attributes, including a large surface area, low weight and excellent thermal as well as mechanical properties [12,13].

Polysaccharides are natural polymeric biomaterials that have been widely used in biotechnological fields due to their availability, biocompatibility and biodegradability [14,15]. Common examples of polysaccharides are starch, cellulose, chitin and chitosan [16]. They can also be modified either chemically or enzymatically for any specific end use. Recently, chitin and chitosan have been used in various applications, including food and nutrition, pharmaceuticals, and biotechnology [17,18]. Owing to its biocompatibility, chitosan has also been deemed a suitable material for wastewater treatment, as well as for medicinal and electrochemical applications [19,20]. As nanotechnology advances, the fabrication of chitosan nanocomposites with organic and inorganic nanofillers has significantly improved the material’s mechanical, chemical and barrier properties [21]. These remarkable results are squandered as there are no works in the literature that study systematic nanostructures, novel bio-nanocomposite possibilities and their associated fabrication processes, which are needed. As a result, this study aims to bridge that gap by presenting future trends as well as challenges associated with chitin and chitosan as a matrix for bio-nanocomposites.

Over the past few years, chitin- and chitosan-derived nanocomposites have shown a tendency towards a wide range of applications. Recently, chitin- and chitosan-based nanomaterials and their remarkable role as dye adsorbents [22], drug delivery vehicles to combat COVID-19 [23], food packaging [24], membranes [25], wound dressings [26] and for biomedical and environmental applications [27,28] have been presented.

To the best of our knowledge, there have been very limited studies which have summarized, in particular, chitin- and chitosan-based nanocomposites for energy-related applications. This work is briefly organized into the background of graphene and graphene oxide (GO) properties as well as the structural properties of chitin/chitosan. In addition, common methods for the fabrication of chitin- and chitosan-derived graphene bio-nanocomposites have been discussed. Finally, it reveals vital information of chitin/chitosan bio-nanocomposites for applications in electronic devices and energy systems. To make it more instructive, this study also presents future recommendations and challenges of current times.

## 2. Graphene and Graphene Oxide

Research related to compatible two-dimensional (2D) CNMs, such as graphite and diamonds, has expanded [29]. Graphene, which is derived from graphite, has been used in a variety of applications. Single-layer graphene was explored theoretically by P. R. Wallace in 1947. It was first unambiguously produced and identified in 2004 [30,31]. Many efforts have been made to mass-produce graphene in selected industries, particularly materials science and chemistry [32]. According to recent price checks, allocated budgets for graphene fabrication and the production of its derivatives reached $67 million in 2015, and are estimated to increase to $680 million by 2020 [33]. Graphene is composed of a single layer of hexagonal graphite with sp^2^-hybridized carbons and sigma connections [34]. Furthermore, delocalized π-type bonds are formed from the remaining p or σ orbitals. It has a two-dimensional structure composed of a layer of carbon atoms that are covalently connected in the form of hexagonal lattices [35]. This is the fundamental structure of many carbon allotropes [36].

**Figure 1:**
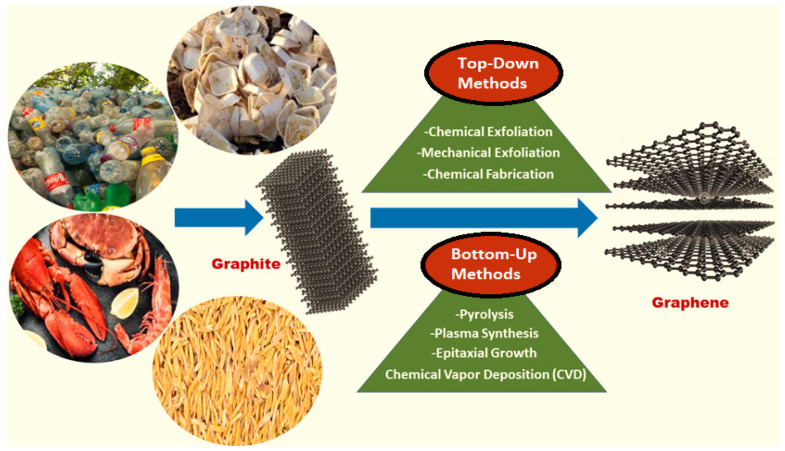
Schematic illustration of graphene synthetic routes from waste sources.

Graphene possesses unique properties, such as a 1.42 Å carbon–carbon connection interval, a 3.3 Å thickness, a large surface area, high movement ability and significant optical, mechanical, electrical and thermal properties [37,38]. Meanwhile, GO was produced by the oxidation of graphite, which is composed of graphene and other functional groups, such as –C=O, –OH, –COOH and –COC– [39]. The existence of oxygenated functional groups on the surface of GO has caused it to have higher capacitance than graphene despite having a smaller surface area [40]. Both GO and reduced graphene oxide (rGO) have great potential to be used in energy-related applications due to their high capacitance, impressive efficiency and enhanced properties as compared to graphene [41,42]. A wide range of synthetic methods have been utilized to convert graphene from waste materials, which are presented in Figure 1.

### Chitin and Chitosan; Structural Analysis

**Figure 2:**
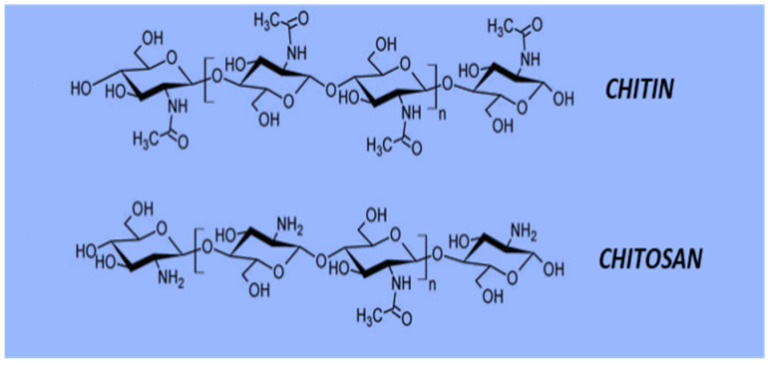
Structural analysis of chitin and chitosan. Reproduced with permission from [53].

Chitosan, the world’s second most abundant biopolymer [43], is composed of N-acetyl glucosamine and glucosamine residues [44]. It is a valuable polymer as it can be easily obtained from marine wastes, including crustaceans and microorganisms such as fungi [45]. Chitosan can be produced in a variety of molecular weights (MWs) and degrees of de-acetylation (DA). Following the DA process, chitosan has been extracted from the solution in the form of powder, fiber and sponges [46]. The solubility of chitosan has a large influence on the ionic concentration, MW, pH, acid nature, DA and distribution of acetyl groups, as well as the main chain. Chitosan is usually dissolved in weak acids, most notably 1% of 0.1 M acetic acid [47]. Furthermore, chitosan can be dissolved in water in the presence of glycerol 2-phosphate at a neutral pH [48]. This type of chitosan is appropriate for plant-based applications [49]. A stable solution can be obtained at room temperature. On the other hand, it endorses reversible gel formation above 40 °C. In comparison to chitin, chitosan possesses better complex-forming ability, which has been attributed primarily to the existing free –NH_2_ groups distributed along its main chain (Figure 2) [50]. Chitosan, a partially deacetylated chitin product, is a copolymer composed of β-(1→4)-2-acetamido-D-glucose and β-(1→4)-2-amino-D-glucose units [51]. In chitosan structure, the R1, R2 and R3 radicals correspond to hydrogen available in plain chitin and chitosan molecules. These surface groups result in the formation of hydroxyl (OH) and amino (NH_2_) groups. They are accountable for the organic modifications of chitosan, which have the potential to produce polymeric derivatives of these compounds [52].

**Figure 3:**
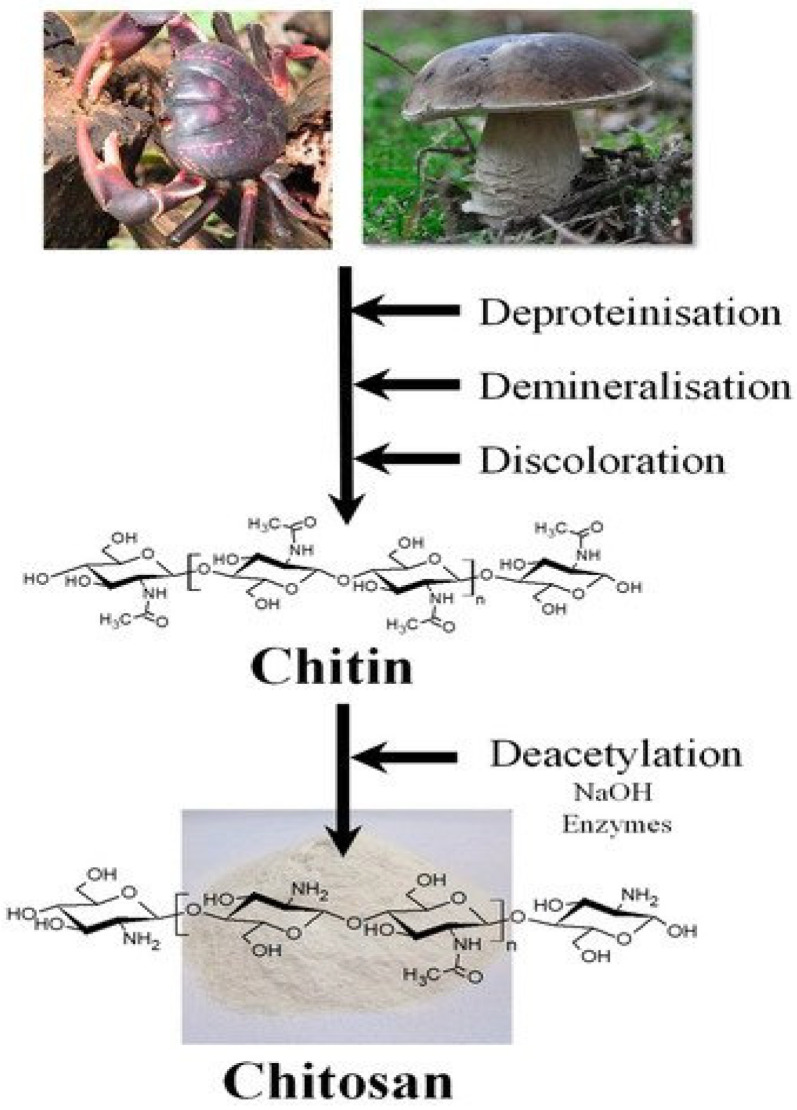
Mechanism of crustacean shell waste conversion into chitin and chitosan [53].

Crustacean shell wastes are a source of biomass raw material for chitin and chitosan production. They contain chitin, lipids, inorganic salts and proteins, as displayed in Figure 3 [54]. Numerous characterization techniques, such as SEM, FTIR, DSC, TGA, 1H liquid-state NMR, XRD and elemental analysis have been used to investigate morphological, structural, degree of DA, crystallinity and other physicochemical or thermal properties [50,55].

## 3. Fabrication of Graphene Nanocomposites

**Figure 4:**
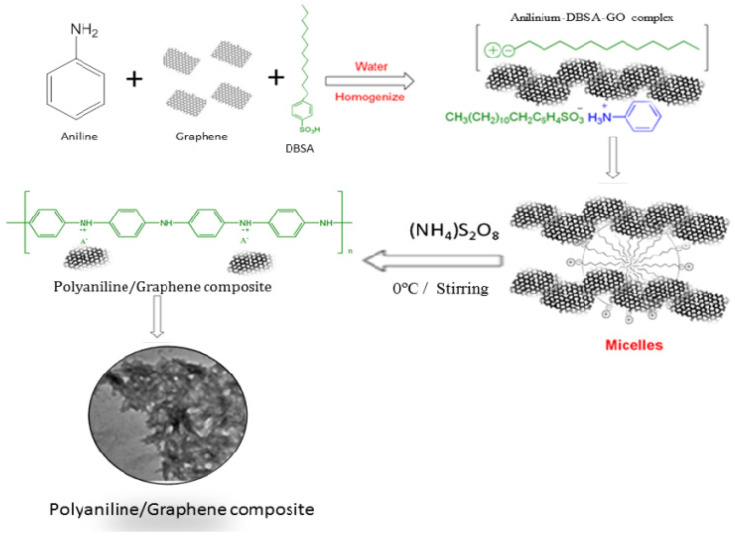
Fabrication of a polymer-based (polyaniline) graphene nanocomposite [61].

The distribution of materials in polymeric matrices has a significant influence on the mechanical, thermal and electrical properties of nanocomposites, as well as their water vapor permeability [56]. Poor distribution of biopolymer–graphene/GO produces unstable nanocomposites, and jeopardizes their properties. The aggregation of graphene/GO nanoparticles and restacking into biopolymeric materials has been a concerning issue [57]. For example, water-soluble polymers such as poly(vinyl alcohol) and poly(ethylene oxide) have been used in the fabrication of GO nanocomposites [58]. Earlier studies have revealed that graphene/GO fillers were incorporated into polymer matrices through common methods, such as solution intercalation, electrospinning and in situ intercalative polymerization, as shown in Figure 4 [59,60].

Most researchers have used a solution intercalation method, in which the chemical structure of a polymer matrix changed as the amount of graphene/GO increased [62]. This was attributed to the occurrence of mild chemical reactions, primarily physical interactions between biopolymers and graphene/GO [60]. Characteristically, this method involves shear mixing of colloidal graphene/GO suspensions with polymers, followed by solvent evaporation. As a result, the adsorbed polymer reassembled, forming a sandwich between the polymer and graphene/GO [63]. Furthermore, polymer–graphene/GO matrices were used in solution-based methods with non-water-soluble polymers via the chemical modification of GO [64]. Poly(methyl methacrylate) and polyurethanes are two examples of non-soluble polymers that have been used in this method [65]. Another interesting method of preparing graphene/GO bio-nanocomposites is in situ polymerization, which uses solvents to lower the dispersions’ viscosity [66]. For example, GO has prepared nanocomposites with enhanced properties by intercalative polymerization of poly(methylmethacrylate) [67] and epoxy resins [68]. Furthermore, polyethylene [69] and polypropylene matrix GO nanocomposites [70] have been successfully prepared via in situ polymerization.

Poly(methylmethacrylate) chains were grafted onto GO to make the filler compatible with the polymeric matrix [71]. The melt-blending method has been used to disperse thermally reduced GO into polymers as well as into a renewable polylactide [72]. Satisfactory distribution rates are attainable through this process. However, it increases polymer melt viscosity, which complicates the process. The preparation of polymer composites, such as graphite, into polypropylene has been done by solid-state shear dispersion using a modified twin-screw extruder [73]. Table 1 entails the synthesis of graphene nanocomposites, comprising polymeric and other matrices, for an enormous number of applications.

### Chitin and Chitosan Graphene Bio-Nanocomposites

GO is an oxidized and hydrophilic form of graphene which greatly enhances functionalities of polymeric matrices [99]. Both graphene and GO are commonly utilized as a nanofiller in polymeric nanocomposites. Chemical modification of graphene has resulted in high-performance nanocomposites with enriched characteristics [100]. Several techniques have been employed to evenly disseminate graphene or GO into chitosan matrices by developing physical/chemical linkages [101].

Chitosan is a green, sustainable and low-cost material. Chitosan-derived nanocomposites have captivated the interest of many researchers due to their exceptional chemical and physical properties [102]. Advances in nanotechnology has led to nanoparticles being deposited on the surface of chitosan. Apart from being used as a pure matrix biomaterial, other nanoparticles have been embedded in bulk materials. Due to chitosan’s hydroxyl (OH) and amine (NH_2_) surface groups, it encourages several inter- and intramolecular hydrogen bond formations [103]. It allows the usage of inorganic and organic fillers, which improves functionalization [104].

Some researchers have doped graphene/GO bio-nanocomposites with starch, chitosan, cellulose and poly(hydroxyalkanoates) [105]. New techniques, such as emulsion droplet coalescence, sieving and spray drying have been applied for the fabrication of chitosan-based products, which are primarily used in medical and pharmaceutical applications [106,107]. The development of new nanocomposite materials continues to be hampered for agricultural applications; however, it is feasible if the source material is equally inexpensive and compatible. Nonetheless, due to chitosan’s properties, it has been used in the fabrication of suitable nanocomposites [108].

Ionic gelation and spray drying are regarded as the best protocols for large-scale CNM production [109]. The ionic gelation method involves the interaction of positively charged chitosan amino groups with negatively charged tripolyphosphate (TTP). TTP is an anionic crosslinker that forms nanoparticles by interacting with chitosan molecules. It is non-toxic and devoid of any innate biological activity. As a result, it is widely used in the production of CNMs [110]. The resulting nanocomposites have been used for foliar applications.

According to a study on the chitosan–rGO nanocomposite, rGO was incorporated into the chitosan crystalline network to improve the adsorption and dye attraction properties of fabricated nanocomposites [111]. At 6 wt.% of rGO nanocomposite, tensile strength, Young’s modulus, elongation at break and conductivity values were increased. However, aggregation occurred at 7 wt.% of rGO, and nanocomposite film conductivity was decreased [112].

**Figure 5:**
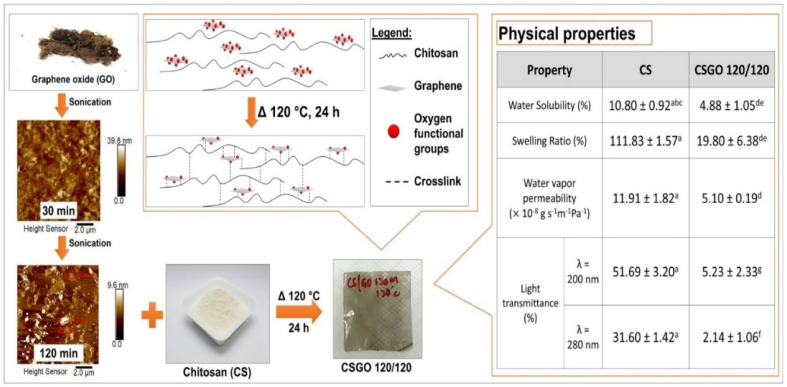
Fabrication of chitosan/GO nanocomposite via ultrasonication which controlled the size and thickness of GO nanosheets. The nanocomposite thin film showed a remarkable decline in UV transmittance, a temperature of 120 °C and compacted hydrophilic properties [116].

Another study on the chitosan-GO nanocomposite reported the existence of a strong interaction between GO and chitosan [113]. An FTIR analysis revealed two peaks, one of which was the amine stretch of chitosan, and the other belonged to the OH group of GO. The fabricated nanocomposites showed properties that were identical to chitosan and GO. Peaks associated with the C=C bonds of GO moved to lower wavenumbers. It occurred due to hydrogen bonding between GO and the chitosan network [114]. In the XRD study, GO was completely exfoliated when the diffraction angles of the chitosan-GO composite were similar to those of the chitosan film. Furthermore, the presence of GO in the composite resulted in lower crystallinity due to a longer combination period [115]. Figure 5 represents the fabrication of the chitosan/GO nanocomposite through ultrasonication for 30 and 120 min, followed by AFM morphological analysis. The cross-linkage between chitosan and GO has been evaluated along with their physical properties [116].

**Figure 6:**
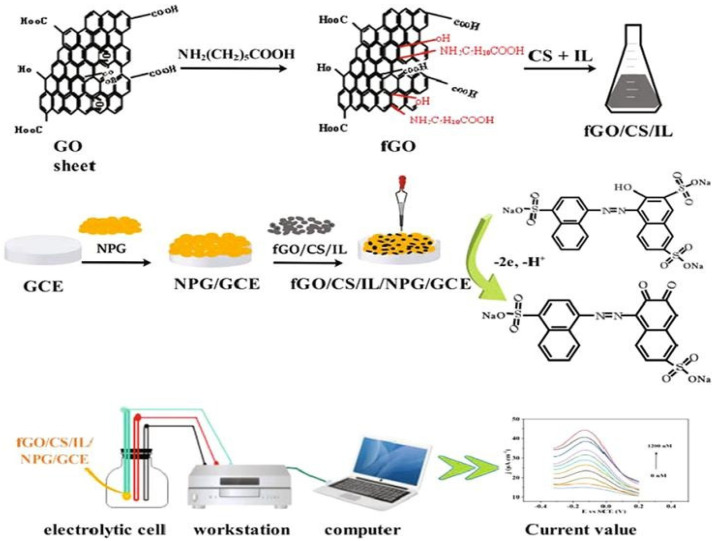
Schematic analysis of novel electrochemical sensor prepared by fGO/chitosan/ionic liquid nanocomposite [120].

Cobos et al. prepared free-standing chitosan–GO nanocomposite films [117]. During the process, GO was dispersed homogenously in chitosan due to the amide linkage formation between carboxylic acid groups of GO and amine groups of chitosan. As compared to pure chitosan, the glass transition temperature of the chitosan–graphene nanocomposite was increased from 118 °C to 158 °C [118]. Furthermore, the tensile strength and Young’s modulus of synthesized nanocomposite was increased by 2.5 and 4.6 times, respectively [119]. In a very recent study, Zhang et al. have synthesized functionalized GO (fGO), by combining chitosan and ionic liquid, for use as an electrochemical sensor, which is displayed in Figure 6. This novel electrochemical senor has been successfully utilized for amaranth detection in commercial beverages, with excellent results [120].

## 4. Energetic Applications of Chitin and Chitosan

The enhanced properties of polymeric materials with metal oxide nanoparticles into composite electrodes have been optimized through the long, linear backbones of chitin and chitosan for electronic devices [121]. This type of synthesis has been conducted to create a hierarchical assembly which connects nanoscopic metal oxide particles to the macro-scale structure of chitosan through electrochemical deposition [122]. Further studies are focused on the MW of chitin and chitosan, which has effected the capacitive behavior and cyclic stability on electrodeposited thin films [19,123].

Chitin and chitosan have emerged as significant polymers for the production of soft materials. This is due to the combination of physicochemical characteristics of biopolymers that enables the hierarchical assembly of nano-sized components at different length scales [124]. The characteristics of chitin and chitosan are influenced by the amount of glucosamine repeating units, crystallinity and degree of DA [125].

### 4.1. Electrical Devices

The development of high-performance composite materials has been expedited by incorporating inorganic “nano-fillers” into polymers. Conventionally, the uses of polymer–inorganic nanocomposites include mechanical, optical, catalytic, magnetic, thermal, electrical and electrochemical applications [126,127]. It is highly desired to attain cost-effective and high-tech functionalized electronic devices for commercial uses. Hence, metal-filled polymer composites are seen as a viable option to meet the requirements of future dielectric technologies [128].

**Figure 7:**
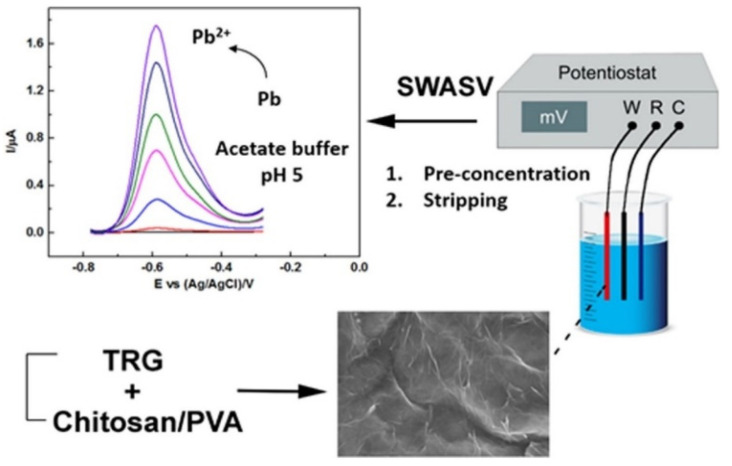
Modification of thermally reduced graphene/chitosan/polyvinyl alcohol nanocomposite as an electrode for Pb(II) detection in wastewater [132].

Nanoparticles are engineered to sustain high electron mobility in order to achieve fast field responses with extraordinary dielectric constants and minimal losses in high-frequency applications [129]. The engineered bio-nanocomposite dielectrics have higher dielectric constants at elevated frequencies and can process polymers at low temperatures as compared to other conventional materials (Figure 7) [130]. Chitosan is preferred in current research prospects due to its ease of accessibility, low cost, environmental friendliness and outstanding mechanical characteristics [131].

In recent years, CNMs such as carbon nanotubes (CNTs) and graphite have been tremendously used among several electronic devices [133]. Metal oxides, such as ZnO, SiO, NiO and TiO_2,_ are typically doped as inorganic nano-fillers for electrical and electrochemical purposes [134]. The dielectric characteristics of NiO have been utilized in the form of nano-fillers for a handful of studies. NiO is a Mott-Hubbard insulator with an extremely low conductivity at ambient temperatures, and has a cubic lattice parameter of 0.4177 nm [135]. However, when the size is lowered to nanoscale, the conductivity of NiO increases significantly due to hole hopping associated with Ni^2+^ vacancies. By incorporating these fillers into non-conducting polymers, they have transformed into conductive polymers while preserving their polymeric properties [136].

Nasrollahzadeh et al. studied in great depth the possibilities of chitin and chitosan as length-scale interconnects. Among the linear carbon backbone of biopolymers, chitosan has provided a location for nanocomponents/fillers linkage within the range of 100–101 nm [137]. The biopolymer exhibits self-organizing properties contributed from the chitosan’s stimuli-responsive film and gel-forming characteristics at microscale levels. Therefore, assembly at greater length scales, i.e., 103 nm, is enabled [138]. Furthermore, localized electrical stimulation promotes the formation of chitin- and chitosan-based films and gels. As a result, both the length scale and nanoscale components of chitosan were linked to electrical equipment. Finally, the metal binding capabilities of chitosan enabled linkages through chelation processes [139]. Kamran et al. have discovered acetic-mediated chitosan-based porous CNMs, which resulted in the capture of CO_2_. They also demonstrated an enhanced surface area (4168 m^2^ g^−1^) and highly effective CO_2_ adsorption performance of fabricated nanomaterials [140]. Currently, there are minimal studies in the literature on the electrochemical behavior and energy production of such novel bio-nanocomposites.

### 4.2. Biosensors

**Figure 8:**
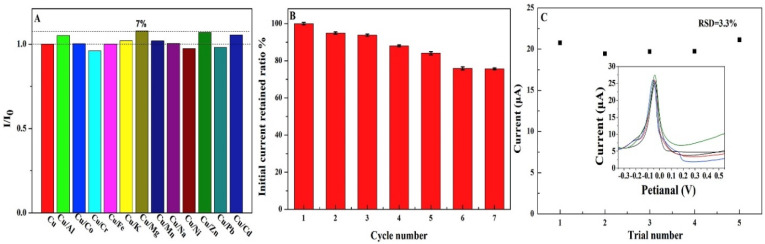
(**A**) Selectivity, (**B**) repeatability and (**C**) reproducibility of chitosan/GO/ion imprinting polymer (CS/GO/IIP) as an electrochemical sensor for Cu ((II) detection (50 μmol/L) [143].

Recently, there has been remarkable use of cost-effective and economic biosensors in energy-related applications [141]. High-quality immobilization of biological recognition components is required to generate dependable biosensors. Chitosan and its bio-nanocomposites have been introduced as effective immobilization matrix materials. Thus, development of novel devices for early-stage illness diagnosis and biomarker detection were possible through these chitosan-bio-nanocomposites-based biosensors (Figure 8) [142].

Fartas et al. have employed graphene/gold nanoparticle/chitosan (GAuCS) nanocomposite films for glucose biosensing. The sensor was designed to immobilize glucose oxidase in thin films of GAuCS nanocomposites at gold electrodes [144]. Likewise, Casteleijn et al. used a simple spin-coating method to modify chitin-based biosensors on Au nanoparticles and polystyrene (PS). Due to chitin’s solubility, this method opened a new domain of future possibilities [145]. The substantial binding of chitin has encouraged strong functionalization for the fabrication of biosensors. Hence, scientists have developed a novel Co^2+^-metal-ion-based plasmon resonance surface sensor through chitin composites for enhanced sensitivity detection [146].

Besides, Ali et al. created a low-temperature H_2_S gas sensor using a conductive chitosan–CuO hybrid nanocomposite at different concentrations ranging from 1–9% volume/volume (*v*/*v*). The chitosan bio-nanocomposite resulted in a sufficiently flexible and transparent semiconductor. The detecting mechanism of the sensor occurs from proton transfer between the gas molecules and amino groups in the chitosan molecule. The presence of glycerol, i.e., OH groups, enhanced the formation of H-bonding [147].

Borgohain et al. have created a pollution sensor that helped to detect Zn^2+^ and Cu^2+^ ions at different concentrations in water. The co-precipitation method was utilized to make chitosan-ZnS quantum dot (QD) sensors. The formation of chitosan-ZnS QD tiny clusters was dependent on the concentration of metal ions in water, with a directly proportional relationship. As a result of the aggregated clusters, the absorption maxima occurred at longer wavelengths, and resulted in reduced energy [148].

### 4.3. Batteries and Electrochemistry

LIBs have become important energy storage systems, especially in the usage of portable electronic devices. Their excellent properties, such as high energy density and low self-discharge rates, are repeatedly noticed [149]. As a result, comprehensive studies on novel electrode materials which are compatible with LIB electrolytes are highly crucial in the advancement of technologies [150].

Using a simple chitosan-assisted hydrothermal and subsequent calcination methods, Ma et al. have developed ultrathin MoS_2_/graphene hetero-structures with high specific surface areas and efficient electrochemical characteristics [151]. Similarly, Chen et al. have introduced a N-doped carbon composite as a cathode material and CNT/chitosan as an amplified separator for innovative lithium–sulfur batteries (LSBs). This simple and effective method is expected to represent a watershed moment for the large-scale manufacturing of hetero-structures with a wide range of applications in batteries [152]. Moreover, Kim et al. have utilized a rGO/chitosan-based binder which has allowed significant improvement in the cycle stability and capacity of LSBs [153].

**Figure 9:**
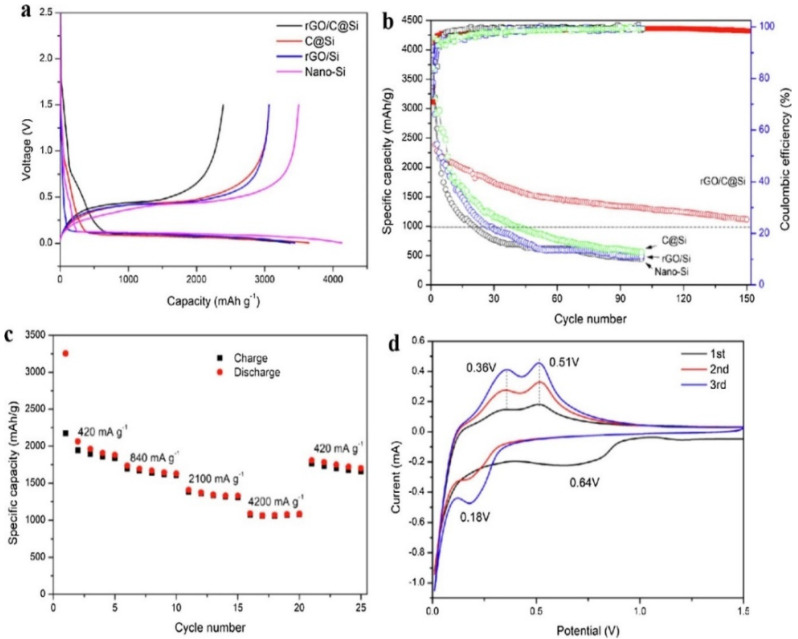
Electrochemical evaluations of fabricated anodes. (**a**) Initial charge/discharge of nano-Si, C@Si, rGO/Si and rGO/C@Si nanocomposites at a current density of 100 mA g^−1^, (**b**) consistent current charge/discharge of nanocomposites and (**c**,**d**) rate performance and current/voltage profile of GO/C@Si anode [158].

Other noteworthy and novel fabricated materials with high electrochemical properties among very recent works include a chitosan-solution-based Si@SiO_2_@N-Carbon anode for LIBs [154], flexible chitosan-based carbon membranes as anodes for potassium- and sodium-ion batteries (KIBs/SIBs) [155], a chitosan-based N-doped carbon/Li_2_ZnTi_3_O_8_/TiO_2_ composite as an anode for LIBs [156], a chitosan-based C@V_2_O_5_ cathode for Zn-ion batteries (ZIBs) [157], a chitosan-based N-doped rGO/C@Si composite for LIBs as shown in Figure 9 [158] and novel S/Se/C supported by a chitosan-based interconnect with CNTs for novel lithium-chalcogenide batteries (NCBs) [159].

### 4.4. Fuel Cells

Fuel cells modified with membranes via proton exchange have been displayed as a promising alternative in eco-friendly energy-related fields. The development of a highly conductive proton membrane is the most important aspect that determines the performance and efficiency of fuel cells [160]. To degrade chitin anaerobically, Li et al. created a microbial fuel cell using *Aeromonas hydrophila*. It was observed that the constructed fuel cell resulted in a seven times faster rate of chitin breakdown as compared to a conventional fermentation system [161]. Yang et al. introduced a low-cost Fe–N–C catalyst, resulting from an Fe(III)-chitosan hydrogel, to improve power generation in microbial fuel cells [162]. Thus, increased power generation was ensured. This approach allowed for the effective breakdown of resistant biomass in order to recover energy [163]. Researchers have reported a chitosan/rGO/polyaniline bio-nanocomposite to be a paradigm bio-anode for glucose-derived fuel cells. It exhibited excellent electrochemical properties with considerable stability [164]. Gorgieva et al. examined an effective chitosan-based N-doped rGO composite membrane for alkaline fuel cells. In this work, chitosan- and graphene-based homogenous materials were engineered using a variety of self-induced methods [165].

Furthermore, a cost-effective method was applied to assemble chitosan/montmorillonite nanocomposite using a ceramic support as an effective membrane for microbial fuel cells [166]. In another study, chitosan was cross-linked to poly(aminoanthraquinone) nanocomposite which was a nitrogen-precursor-based Fe-N-C oxygen reduction catalyst for microbial fuel cells. The high-performance bio-anode in the fuel cell exhibited outstanding shape and retention characteristics due to its synergistic effects between porous structures [167]. This highly porous design along with anode materials have resulted in a 78-fold increase in maximum power density.

**Figure 10:**
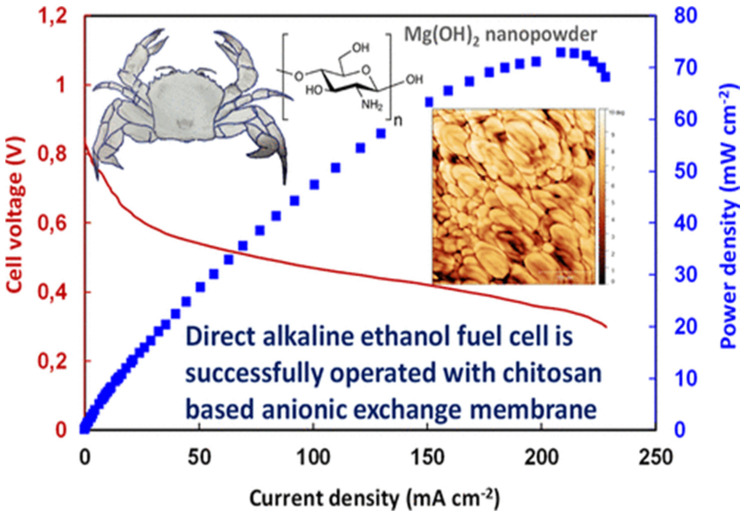
Novel polymer nanocomposite for alkaline fuel cells. Nanocomposite was fabricated using a solvent-casting method in which chitosan, GO, Mg(OH)_2_ and benzyltrimethylammonium chloride were used [169].

Vijayalekshmi et al. created a cross-linked, flexible, oxidative and thermally stable chitosan-based green polymer electrolyte using methane, sulfonic acid and sodium-dodecylbenzene-sulfonic-acid-doped chitosan. At 100 °C, the polymer electrolyte had a conductivity of 4.67 × 10^−4^ S/cm and thermal stability at a maximum value of 260 °C [167]. In short, this low-cost and eco-friendly technique ensures superior methanol barrier performance by reducing methanol absorption at higher methanol concentrations for fuel cells, as displayed in Figure 10 [168].

Tohidian et al. applied a simple sol–gel technique to prepare chitosan-surface-modified CNT bio-nanocomposites. Additional benefits of chitosan-coated CNTs include a reduced danger of electronic short-circuiting and improved interaction between CNTs and chitosan, resulting in uniform dispersion [170]. Compared to pure chitosan membranes, bio-nanocomposites showed better thermal stability, proton conductivity and mechanical characteristics. Due to the electrostatic and hydrogen bonding between molecules, bio-nanocomposites achieved a power density of 98.5 mW cm^−2^ at 70 °C [171]. Conversely, the reduced conductivity of protons was caused by the amino functional group, which led to less water uptake.

### 4.5. Supercapacitors

Supercapacitors are a superior type of energy storage device. They exude higher capacitance, power density, durability cycle and stability. Many studies have been devoted to presenting novel nanomaterials as electrode materials from sustainable resources, due to their impressive electrochemical performance [122,172]. Recent research has been dedicated to produce chitosan-based bio-nanocomposites with remarkable properties, such as high power, an outstanding life cycle and an eco-friendly nature as compared to expensive nanoparticles [173,174].

A three-step technique which included the aerogel synthesis, aerogel carbonization and nitrogen self-doping processes was used to create chitosan-based supercapacitors for elevated enactment. This bio-nanocomposite showed a specific capacitance of 331 F g^−1^ in 6 mol L^−1^ using a KOH electrolyte at 1 A g^−1^, with excellent stability of 90% after 10,000 cycles [175]. Major and noteworthy performance values were reached by preparing chitosan-based supercapacitors, which showed specific energy and power density at maximum recorded values of 10.46 Wh kg^−1^ and 500.08 W kg^−1^, respectively [176]. Similarly, Lin et al. have created a chitosan-based hydrogel using an ultrafast hydrogelation technique in which carboxylation of chitosan was carried out at 6, 8 and 10 values, thus producing a supramolecular electrolyte hydrogel with excellent specific capacitance of chitosan, chitosan-6, chitosan-8 and chitosan-10 supercapacitors at 35 F∙g^−1^, 72.5 F∙g^−1^, 49.2 F∙g^−1^ and 40.5 F∙g^−1^, respectively [177]. It was a facile and simple technology which has shown great potential for developing future bio-based supercapacitors.

### 4.6. Solar Cells

Following cellulose, chitosan is the most abundant biomass-containing amino-polysaccharide on earth. The ability of chitosan to produce a transparent film while maintaining its properties has influenced scientists’ interest in designing solar cells [178]. The amine groups on the main chain of chitosan make it a viable option for cathode interlayers, while additional functionalization is added due to the hydroxyl and amino groups throughout the main chain [179].

**Figure 11:**
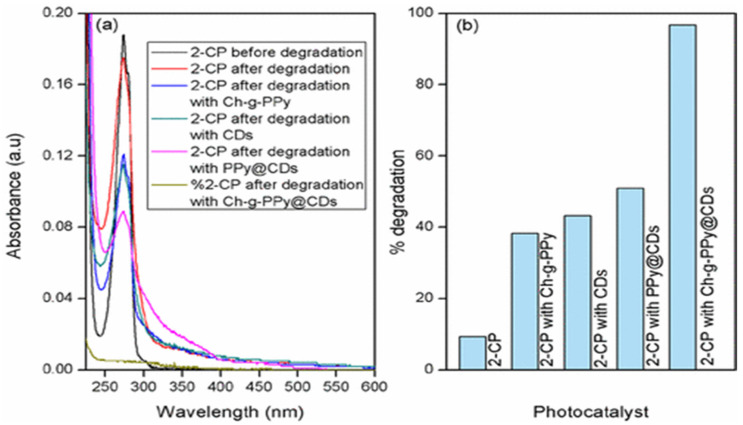
(**a**) Photocatalytic degradation of various nanomaterials via sunlight irradiation after 90 min, (**b**) efficiency of degradation comparing catalysts such as chitosan-grafted polypyrrole (Ch-g-PPy), polyrrole-based carbon dot (PPy@CD) and chiton-grafted polypyrrole carbon dot (Ch-g-PPy@CD) nanocomposites [183].

Praveen et al. produced organic solar cells with a power conversion efficiency of 5.83% by utilizing layer-by-layer and self-assembled chitosan with a uniform and controllable nanoscale thickness [180]. The engineered solar cells exhibited higher efficiency due to their organized structural form, which produced both interfacial and molecule dipoles. The dipoles reduced the work function of electrodes, which has led to their promising and highly compatible utilization [181]. The self-assembled chitosan has improved performance up from 100 to 200% as compared to spin-coated chitosan interlayers and fuel cells. It was observed without any cathode interlayer, in terms of power conversion and efficacy, respectively (Figure 11) [182].

Moreover, Zulkifli et al. used a chitosan-based polymer electrolyte to create a high-performance plasmonic dye-sensitized solar cell [184]. Polyethene oxide (PEO) was placed between the TiO_2_/dye photoelectrode and the Pt counter electrode in a chitosan-based solar cell. A simple solution-casting approach was used to add the ion donor NH4I salt. The mixing of chitosan with PEO was performed, which enhanced the flexibility of the electrolyte, the mobility of ions and the conductivity [185]. Thus, the 16.5 wt.% of chitosan and 38.5 wt.% of PEO:NH4I exhibited a significant increase in conductivity, respectively. The dye-sensitized solar cells (DSSC) recorded a 19-fold improvement of overall efficiency from 0.06% to 1.13% by incorporating TiO_2_ particles into the cell system [186]. Table 2 summarizes chitosan/chitin-based nanomaterials, their mode of fabrication and utilization in energy-related applications.

## 5. Limitations and Challenges

**Figure 12:**
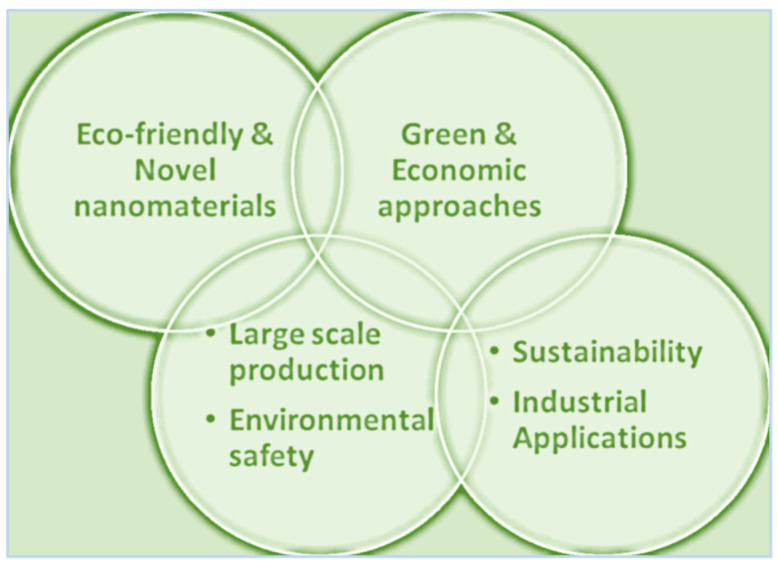
Future perspectives.

The applications and merits of novel and promising biopolymers, i.e., chitin- and chitosan-based bio-nanocomposites are apparent to mitigate future global issues. Despite having outstanding biological and physiochemical characteristics, the molecule’s flaws offer major hurdles and limits to its applicability in a variety of key industries. Therefore, several outlined disadvantages and problems must be addressed to ensure the modification of chitosan-based bio-nanocomposites. There are major thoughtful drawbacks which must be highlighted (Figure 12), and are outlined below:

- The current limitations in the medicinal fields are caused by low solubility and pH, which have led to instable physiological changes among nanocomposites.
- The hygiene and safety of synthesized bio-nanocomposites remain uncertain as the European Food Safety Authority (EFSA) denies them, despite possessing an approval for food contact from the Food Development Authority (FDA).
- Low colloidal stability makes chitin- and chitosan-based bio-nanocomposites unsuitable for large-scale drug delivery.
- Elevated elasticity of chitosan-based bio-nanocomposites restricts their use and applications.
- Despite showing satisfactory effectiveness in several medicinal applications, there are numerous issues such as drug release, loading efficacy and capacity, rate of degradation, and functionalization.
- Finally, industrial processing centers continue to face financial challenges in establishing a solid commercial viability of sustainable biopolymers in the real world.

### Future Recommendations

The importance of chitosan-based nanocomposites is skyrocketing owing to their plentiful advantages. However, it does have some difficulties that must be overcome. The following section narrates some elements and suggestions for future study and research:

- Nanotechnology has great potential in agro-economics to improve agricultural areas. In this regard, nano-chitin or nano-chitosan might be powerful tools for delivering environmentally benign nano-chemicals or nano-agro-fertilizers.
- Their components can be used to grow crops, manage pests, increase fish output, produce meat, preserve seeds, improve the immune system of crops and develop crops with high drought and salinity resistance, among other elements.
- There are very few in vivo studies demonstrating the formulation and conjugation of chitosan-based nano-carriers with antibodies as well as the assessment of long-term toxicity of the nano-carriers. Thus, future research can be conducted on studies of antibodies coupled with chitosan-based nano-carriers.
- As metal oxides have shown unique semiconducting, optical and photocatalytic characteristics, chitosan/metal oxide bio-nanocomposites could bring a remarkable change for wound healing and other future regeneration studies.
- Chitin and chitosan can be an attractive future research choice as heterogeneous bio-nanocatalysts and kinetic studies.
- Due to their physiological pH, chitosan-based bio-nanocomposites have limited solubility. In this situation, researchers should concentrate on developing novel chitosan-based bio-nanocomposite materials with improved solubility and aggregation.
- Conventional acid and alkali treatments should be replaced with novel biological methods for chitosan extraction. In competitive industrial situations, eco-friendly and cost-effective extraction methods must also be established.
- Crabs, shrimps, insects and fungi are acquiring enormous demand in many industrial areas due to their remarkable characteristics. As these natural resources become more popular, there is a growing worry that they will become extinct. The researchers’ mission should be focused on identifying alternate sources of energy in order to restore ecological equilibrium.
- Researchers should concentrate on introducing nanomaterials with high degrees of elasticity for novel electronic devices.

## 6. Conclusions

To summarize, the hazardous impact of toxic and non-biodegradable materials has caused adverse effects on human health and the environment. Consequently, it has indeed seized the attention of researchers to introduce a variety of bio-nanocomposites. Currently, chitin- and chitosan-derived bio-nanocomposites are the emerging alternates with outstanding functional properties and compatibility. Herein, we have summarized their structural analysis and fabrication into a large number of graphene-based bio-nanocomposites. It was observed that several types of fabrication methods have been utilized which impart different properties for their particular applications. In particular, progressive trends of energetic applications have been elaborated with their impressive attributes towards electronic devices. Finally, we conclude that chitosan-derived graphene bio-nanocomposites have a strong potential for unexplored applications, such as wastewater treatment and environmental pollution, as well as within the oil and gas industry, such as for drilling fluids, mechanical operations, kinetic and computational chemistry. 

## Figures and Tables

**Table 1:** Summary of the graphene nanocomposites with inorganic and polymeric materials.

Counterparts	Manufacturing Methods	Parameters and Conditions	Applications	Ref.
** Graphene–Polymer Nanocomposites **				
Three-dimensional graphene-based polymer nanocomposite	Three methods were used; Three-dimensional graphene-based template Polymer particle/foam template Organic-molecule-cross-linked graphene	-	Energy storage and conversion Electromagnetic interference shielding Oil/water separation Sensors	[74]
Polyaniline/GO nanocomposite	Electrospinning technique	Detection limit: 0.01 mg/g	Determination of nicotine	[75]
Polyaniline/GO nanocomposite	Chemical exfoliation	Detection limit: 0.1 µg/L Quantification limit: 0.4 µg/L	As adsorbent in the presence of chelating dithizone ligand to measure cadmium (II) ions in aqueous media	[76]
Hydrogels of conjugate polymer polypyrrole (PPy)/rGO composite	-	Surface area: 21.48 m^2^/g	As heavy metal sensors for simultaneous detection of Cd^2+,^ Pb^2+,^ Cu^2+^ and Hg^2+^	[77]
Polylactic acid (PLA)/GO nanocomposite	Solution blending and coagulation	PLA/GO 1 wt.%	Enhanced mechanical properties	[78]
Polyurethane–GO nanocomposite	-	Thermal stability increased to 217 °C Good electrical conductivity: 1.39 × 10^−9^ Scm^−1^	Sensing material for optical fibers	[79]
Polyaniline nanofibers/functionalized rGO composite films	Hybrid suspension of GO and in situ polymerized polyaniline nanofibers were filtered, followed by hydrothermal treatment	-	Composite films were uniform, flexible and stable High specific capacity: 692 F/g at 1 A/g As electrodes High capacitance of 324.4 F/g at 1 A/g Energy density: 16.3 Wh/kg at power density of 300 W/kg	[80]
Bacterial cellulose/graphene/polyaniline nanocomposite	Two-step strategy	Electrical conductivity: 1.7 ± 0.1 S/cm	Electromagnetic shielding Flexible electrodes	[81]
**Graphene/Activated Carbon Nanocomposites**				
AG/PMB/GS/GCE	Ag nanocrystals were electrodeposited on different polymer dyes, poly (methylene blue) or poly (4-(2-Pyridylazo)-Resorcinol) (PAR)-modified graphene carbon spheres (GS) hybrids	Detection limit: 0.15 µM Sensitivity: 400 µAm/Mcm^2^	Sensor for H_2_O_2_ detection	[82]
rGO/activated carbon nanosheet composite	-	Specific capacitance of the electrode material by 58.2%	High-performance electrode material for supercapacitor	[83]
Glucose-treated rGO–activated carbon (rGO/AC) composites	Hydrothermal technique	Detection of glucose in range of 0.002 to 10 mM Sensitivity: 61.06 µA/mMcm^2^ Response time: 4 s Low detection limit: 2 µM	Biosensor	[84]
**Graphene/Metal Oxide Nanocomposites**				
HBcAG/gold nanoparticles–rGO–enAu nanocomposite	-	Lowest detection limit: 3.8 ng/mL at 3 σ/m	Anti-hepatitis antigen detection	[85]
Fe-doped SnO_2_/rGO nanocomposite	Fe-doped SnO_2_ was hybridized with different iron concentrations and rGOHydrothermal method	-	Photocatalysis	[86]
ZnO–graphene composite	Hydrothermal method	Band gap energy: 2.84 eV Photoluminescence lifetime: 21.60 ns		[87]
TiO_2_/rGO nanocomposite	-	Good catalytic activity Cooking oil converted into biodiesel at a rate of 98%	Heterogeneous catalyst for transesterification of waste cooking oil into biodiesel	[88]
GO–Cu_2_O nanocomposite	-	Agglomerated Cu nanoparticles were distributed uniformly over rGO sheets at 400 °C Electrical conductivity similar to GO monolayer sheets	Supercapacitor	[89]
2D MnO_2_/rGO nanocomposite	Wet chemical method at low temperature	Discharged specific capacity maintains at 242 mAh/g after 60 cycles at 0.1 C	Cathode material of lithium-ion batteries (LIBs)	[90]
rGO/silver nanowires (AgNWs)/Ga-doped zinc oxide (GZO) composite thin films	-	Excellent electrical conductivity Superior stability to a mono/bilayer of electrodes Resistance increased to less than 5% when exposed to atmosphere for 60 days	Composite electrode	[91]
rGO/CuO nanocomposite	Impregnation of microsized malachite spheres on GO sheets followed by calcination at 300–500 °C for 5 h	Efficient nanocatalysts compared to CuO nanoparticles	Catalyst	[92]
3D NiO hollow sphere/rGO composite	Coordinating etching and precipitating process by using Cu_2_O nanosphere/GO composite as a template	Sensitivity: 2.04 mA mM^−1^cm^−2^ Response time: 5 s Good stability	Glucose sensor	[93]
Fe_2_O_3_/rGO composite	Hydrothermal method	Specific discharge battery: 1366 mAh/g at 0.1 A/g (LIBs) and 318.9 F/g at 0.1 A/g	Electrode material for supercapacitor Anode material for LIBs	[94]
**Graphene/Metal Nanocomposites**				
rGO/Co_9_S_8_ composites	-	High discharge capacity: 551 mAh/g at 0.1 A/g Good rate capability at 10 A/g	Advanced sodium-ion battery anode Na_3_V_2_(PO_4_)_3_krGO/Co_9_S_8_ full cells	[95]
Three-dimensional porous-laser-induced graphene–silver nanocomposite	-	High uniform electrical conductivity Low detection limit: 5 µM	Glucose sensor	[96]
Nitrogen-doped graphene–copper nanocomposite		Electrical resistivity: 0.16 µΩ cm at room temperature Thermal conductivity: 538 W/mK at 25 °C	High thermal conductivity	[97]
SH-β-CD-rGO/Cu nanospheres nanocomposite	Chemical deposition of Cu nanospheres on SH-β-CD-rGO	Good sensitivity Low detection limit: 20 nM	Used for rapid and sensitive electrochemical method to determine trace 4-NP in water	[98]

**Table 2:** Tabulation of chitin/chitosan-based nanomaterials and their energetic applications.

Composite Materials	Manufacturing Routes	Applications	Outcomes	Ref.
Semiconducting chitosan film	Casting method	H_2_S gas sensor	Average min. response time of 14.9 ± 3.7 s with a 15 ppm detection limit	[147,187]
Fe_3_O_4_/chitosan	Chemical modification	Biosensor for gallic acid (GA) detection	Detection limit of 12.1 nM Dynamic range of 0.5–300.0 μm	[188]
Ti–6Al–4V alloy coated with fumed silica/chitosan/poly(vinylpyrrolidone) composite	Artificial saliva solution	Coating for electrochemical corrosion	Inhibition efficiency of 99.85%	[189]
Fe/chitosan-coated carbon electrode	Co-electrodeposition	Sensor for As(III) detection	Recorded detection limits of 1.12 ppb and 1.01 ppb for mining wastewater and soil, respectively	[190]
Ag nanoparticles/chitosan-thiourea-formaldehyde	Polymeric metal complexation	Biosensor for non-enzymatic glucose detection	Detection limit of 0.046 mM with 35.22 mA mM^−1^ cm^−2^ sensitivity	[191]
F-rGO @ CNTs/chitosan	Freeze-drying and dip-coating	Piezoresistive pressure sensor	Response time of 170 ms and sensitivities of 4.97 kPa^−1^ and 0.05 kPa^−1^ in 0–3 kPa and 40–80 kPa, respectively	[192]
Chitosan/zinc oxide/single-walled CNTs	Solution casting	Chemiresistive humidity sensor	Range of humidity detection of 11–97%	[193]
Copper ferrite nanoparticles/chitosan	Ultra-sonication	High-performance electrochemical	Range of detection limit between 0.025–697.175 μM	[194]
Localized surface plasmon resonance (LSPR)-based optical fiber/chitosan-capped gold nanoparticles on BSA	Chemical modification	Optical fiber sensor for Hg(II) detection	Limits of detection for water and seawater were 0.1 and 0.2 ppb, respectively	[195]
Graphene QDs/chitosan	Ultrasound dispersion	Humidity sensor	High response sensitivity and short response/recovery time, i.e., 36 s/3 s	[196]
Polypyrrole/chitin nanofibers/carbon nanotubes	Vacuum filtration with freeze-drying	Supercapacitors	2 Ag^−1^ records an 86.6% retention rate after 5000 cycles and 362 F g^−1^ specific capacity at 5 mVs^−1^ in 1 molL^−1^	[197]
Chitin/GO/zinc oxide/polyaniline	Co-polymerisation	Chitin-based polyaniline electrode for Cu(II) detection	Response time of 240 s and detection limit of 13.77 ppm	[198]
Chitosan/cellulose acetate/PVA gel	Phase inversion and polymerisation	Supercapacitors	Retention rate of 71.2% after 1000 cycles Specific capacitance of 5.5 mFcm^−1^ at 20 mVs^−1^	[199]
MOF-5/chitosan	Chemical modification	High-performance supercapacitors	Specific capacitance and capability rate records values of 199.9 F g^−1^ and 75.6%, respectively	[200]
Polyionic liquid/carboxymethyl chitosan	Direct carbonization	Supercapacitors	At 0.1 Ag^−1^, specific capacitance of 633 F g^−1^ and stability after 10,000 cycles	[201]
Chitosan/graphene/ionic liquid/ferrocene nanocomposite	Chemical modification and drop-coating	Electrochemical immunosensor	Highly selective and sensitive to prostate-specific antigen and detection limit of 4.8 × 10^−8^ ng mL^−1^	[202]
Polyaniline-grafted chitosan/GO-CNT/Fe_3_O_4_ nanocomposite	Solution mixing evaporation	Electrode material for supercapacitors	Life cycle of 99.8% Specific capacitance at 100 Ag^−1^	[203]
Nano-cobalt/chitosan composite coating	Implantation	Electrochemical and H_2_ evolution	Resistance value of 1150.77 kΩ cm^2^ Nanocomposite coating up to 99.01%	[204]
Chitin from prawn shell/sodium dihydrogen citrate	Chemical extraction and drying in vacuum conditions	Batteries	The stable discharge capacity of roughly 157 mAh g^−1^ surpassing 15 cycles	[205]
Chitin fiber non-woven separator	Centrifugal jet spinning	Fuel cells	Current discharge at ±200 μA	[206]
Sulfonated chitosan/GO	Casting	Direct methanol fuel cells	Conductivity of 4.86 × 10^−3^ Scm^−1^ at (25 °C), Selectivity of 1.89 × 10^5^ Scm^−3^ s	[207]
Chitosan/GO on membrane substrates of sulfonated poly(vinylidenefluoride)	Sulfonation and alternate dipping	High-temperature proton exchange membrane fuel cells	Conductivity values of 2.34 × 10^−1^ S/cm and 1.56 × 10^−1^ S/cm at 140 °C Methanol permeability was (2.26–3.46) × 10^−7^ cm^2^/s Proton conductivity reached 2.34 × 10^−1^ S/cm at 140 °C under anhydrous conditions	[208]
Chitosan/GO aerogel	Hydrothermal method	Microwave absorption	Density value of 125 cm^2^/g and specific shielding effectiveness of ~−556 dBcm^3^/g	[209]
Chitosan/hydroxyl ethylcellulose/polyaniline loaded with GO doped by silver nanoparticles bio-nanocomposite as a hydrogel	Hydrothermal method	Efficient semiconductor material	Hydrogels improved DC conductivity by about 25 times from 3.37 × 10^−3^ to 8.53 × 10^−2^ S/cm	[210]
Chitosan/ammonium thiocyanate	Solution-casting technique	Electric double-layer capacitor	High ionic conductivity of 8.57 × 10^−4^ S/cm was obtained	[211]

## Data Availability

Not applicable.
